# Cognitive Functioning of Unaffected First-degree Relatives of Individuals With Late-onset Alzheimer's Disease: A Systematic Literature Review and Meta-analysis

**DOI:** 10.1007/s11065-022-09555-2

**Published:** 2022-09-03

**Authors:** Ari Alex Ramos, Noelia Galiano-Castillo, Liana Machado

**Affiliations:** 1 Department of Psychology and Brain Health Research Centre, University of Otago, Dunedin, New Zealand; 2 Brain Research New Zealand, Auckland, New Zealand; 3 Department of Physical Therapy, Health Sciences Faculty, “Cuidate” from Biomedical Group (BIO277), Instituto de Investigación Biosanitaria (ibs.GRANADA), and Sport and Health Research Center (IMUDs), Granada, Spain, University of Granada, Granada, Spain; 4 Department of Psychology, Pontifical Catholic University of Paraná, Rua Imaculada Conceição, 1155, Curitiba, CEP 80.215-901 Brazil

**Keywords:** Alzheimer disease, APOE ɛ4, Cognitive dysfunction, Neuropsychological tests, Risk factors, Family history

## Abstract

First-degree relatives of individuals with late-onset Alzheimer's disease (LOAD) are at increased risk for developing dementia, yet the associations between family history of LOAD and cognitive dysfunction remain unclear. In this quantitative review, we provide the first meta-analysis on the cognitive profile of unaffected first-degree blood relatives of LOAD-affected individuals compared to controls without a family history of LOAD. A systematic literature search was conducted in PsycINFO, PubMed /MEDLINE, and Scopus. We fitted a three-level structural equation modeling meta-analysis to control for non-independent effect sizes. Heterogeneity and risk of publication bias were also investigated. Thirty-four studies enabled us to estimate 218 effect sizes across several cognitive domains. Overall, first-degree relatives (*n* = 4,086, mean age = 57.40, *SD* = 4.71) showed significantly inferior cognitive performance (Hedges’ *g* = -0.16; 95% CI, -0.25 to -0.08; *p* < .001) compared to controls (*n* = 2,388, mean age = 58.43, *SD* = 5.69). Specifically, controls outperformed first-degree relatives in language, visuospatial and verbal long-term memory, executive functions, verbal short-term memory, and verbal IQ. Among the first-degree relatives, APOE ɛ4 carriership was associated with more significant dysfunction in cognition (*g* = -0.24; 95% CI, -0.38 to -0.11; *p* < .001) compared to non-carriers (*g* = -0.14; 95% CI, -0.28 to -0.01; *p* = .04). Cognitive test type was significantly associated with between-group differences, accounting for 65% (*R*^2^_3_ = .6499) of the effect size heterogeneity in the fitted regression model. No evidence of publication bias was found. The current findings provide support for mild but robust cognitive dysfunction in first-degree relatives of LOAD-affected individuals that appears to be moderated by cognitive domain, cognitive test type, and APOE ɛ4.

## Introduction

Family studies have indicated that first-degree relatives of individuals with late-onset Alzheimer's disease (LOAD) are at increased risk for developing dementia (Cannon-Albright et al., 2019; Cupples et al., 2004; Silverman et al., 1994). In addition, previous studies have shown that the odds of developing dementia in first-degree relatives of individuals suffering from LOAD is between 2.9 and 6.1 times that of first-degree relatives without a family history of LOAD (Mayeux et al., 1991; Scarabino et al., 2016). Of note, offspring of individuals with LOAD tend to show decreased brain metabolism in the same areas affected by clinical LOAD, such as posterior cingulate, precuneus, medial temporal, and parietal cortex (Donix et al., 2010; Mosconi et al., 2013, 2014; Okonkwo et al., 2012). Since LOAD-related neuropathological changes precede the clinical diagnosis of LOAD by many years (Sperling et al., 2011), identifying early subtle signs of cognitive decline in unaffected first-degree relatives is of paramount importance to developing effective preventive interventions to delay progression to dementia.

To date, neuropsychological findings regarding the cognitive profile of first-degree relatives of individuals with LOAD are inconsistent. For instance, while some research has shown decreased executive function (Abulafia et al., 2019a, b; Donix et al., 2012; Hazlett et al., 2015) and poorer memory recall (Abulafia et al., 2019a, b; Duarte-Abritta et al., 2018; Rice et al., 2003) in first-degree relatives compared to controls without a family history of LOAD, other studies have failed to find significant performance differences on neuropsychological tests (Donix et al., 2010; Ercoli et al., 2005; Johnson et al., 2006; McPherson et al., 1995; Miller et al., 2005; Ritchie et al., 2017). Given that LOAD is a complex neurological disorder, several factors could contribute to these seemingly contradictory findings. In the present quantitative review, we considered two potentially important variables, namely, age and the ε4 allele of the apolipoprotein E gene (APOE ε4).

Aging-associated morphological and functional changes in brain cells (e.g., astrocytes, microglia, and neurons) lead to older age being the major known risk factor for neurodegenerative diseases (Behfar et al., 2022; Hullinger & Puglielli, 2017). For instance, animal studies suggest loss of synapses and dendritic spines and decreased neurogenesis characterize brain aging (Geinisman et al., 1992; Hamilton et al., 2013; Pannese, 2011; Peters et al., 2008; Rybka et al., 2019). In particular, clinical signs of LOAD-related cognitive impairments usually appear by 65 years and may reflect shortcomings in the individual’s brain to successfully adapt to changes associated with aging (Mecocci et al., 2018). Interestingly, a recent web-based study with a large sample of self-reported first-degree relatives (*n* = 59,571) of individuals with LOAD found that performance on a verbal paired-associates learning task decreased by a rate of two word-pairs per decade of life (Talboom et al., 2019). In addition, an investigation of cognitive performance differences of 168 family members (e.g., offspring, siblings, grandchildren) of nine LOAD-affected individuals against 187 controls without a family history of LOAD provided evidence of significant group differences only in family members aged 70 years or more (Zeng et al., 2013). Although the latter study was not focused only on first-degree relatives, collectively these findings suggest that age may have a significant influence on cognitive performance differences between first-degree relatives and controls, thus warranting investigation here.

Accumulating evidence from the last 30 years has supported the APOE ε4 allele as the major single genetic risk factor for LOAD (Gottschalk et al., 2016; Liu et al., 2013; Yang et al., 2021), and the development of drugs and other interventions aimed at reducing the adverse impact of APOE ε4 is currently deemed a promising avenue for treating LOAD (Martens et al., 2022; Yang et al., 2021). Importantly, a previous survival analysis of six population-based studies showed that carrying the APOE ε4 variant is associated with increased risk of mortality (Wolters et al., 2019). In addition, in individuals with accumulation of amyloid β (Aβ) peptides, a neuropathological hallmark of LOAD, the prevalence of APOE ε4 is higher in those with mild cognitive impairment (63.5%) or LOAD (66.1%) compared to cognitively normal peers (50.9%). Together, these findings signal the importance of gaining more information about the influence of APOE ε4 on cognition in first-degree relatives of LOAD-affected individuals. This is underscored by the general population prevalence of ε4 carriers being estimated at 14% (ALZGENE, 2010), whereas the Wisconsin Registry for Alzheimer's Prevention (Johnson et al., 2018) and the Israel Registry for Alzheimer's Prevention (Ravona-Springer et al., 2020), two independent ongoing longitudinal studies on risk factors for LOAD, both showed almost 50% of adult children of LOAD-affected individuals are ɛ4 carriers (Eisenberg et al., 2010; Wolters et al., 2019). Furthermore, Yi et al. (2018) recently found that having a first-degree family history of LOAD and carrying the APOE ε4 allele are synergistically associated with higher Aβ deposition and reduced regional cerebral glucose metabolism in voxel-wise analyses. However, although APOE ε4 may play a role in cognitive dysfunction in unaffected relatives (Donix et al., 2012), no previous study has synthesized statistical data to investigate the association between the APOE ε4 genotype and cognitive dysfunction in first-degree relatives.

Given that the sample size of most studies on this topic is small, and the cognitive tests and relevant domains have varied across studies, interpretation of the existing data is in need of a meta-analytical review to increase statistical power and provide a more reliable estimation of the population effect size. Thus, the purpose of this quantitative synthesis was twofold. First, we sought to investigate the association between family history of LOAD and cognitive functioning by means of a meta-analysis quantifying performance differences of unaffected first-degree relatives (e.g., sibling or offspring) compared to controls without a family history of LOAD. Second, we endeavored to explore potential moderator variables of effect size heterogeneity that may help account for the seemingly contradictory research outcomes pertaining to the impact of family history of LOAD on cognition.

### Methods

A comprehensive literature search was initially undertaken on October 10, 2019, using PsycINFO and Web of Science databases with no imposed timeframe restriction. This initial search identified 5,678 records, of which 29 were deemed relevant for the current meta-analysis. Based on information from these 29 records, we carried out a second systematic search on November 3, 2020, using specific search terms (e.g., medical subject headings) in PsycINFO, PubMed/MEDLINE, and Scopus (for details about search terms and strings for each database, see Appendix of the Supplementary Online Content). The overall literature search, supplemented by manually searching the reference lists of articles deemed relevant, provided a final sample of 34 articles that met the eligibility criteria and were included in the meta-analysis. Note that we contacted five corresponding authors to request the statistics not reported in their articles required to estimate effect sizes, but only one provided the missing statistics. As a result, the remaining articles with missing statistics were excluded. Duplicate references were removed using EndNote. The current study followed the Preferred Reporting Items for Systematic Reviews and Meta-Analyses guidelines (Page et al., 2021).

### Eligibility Criteria and Outcome

The meta-analysis included published empirical studies that provided cognitive test results comparing first-degree relatives of individuals with LOAD against a group without a family history of LOAD. Only participants categorized as cognitively healthy (i.e., no diagnosis of cognitive impairment) were included. The criteria used to exclude studies included first-degree relatives of individuals with autosomal dominantly inherited familial AD, also known as early-onset AD (Bertram & Tanzi, 2008), first-degree relatives of individuals with non-LOAD dementia type (e.g., frontotemporal dementia), lack of required statistics (e.g., mean and standard deviation, *t* test or *F* test results) to compute effect sizes, and theoretical studies, commentaries, letters to the editor, case studies, and conference proceedings. In cases of duplicate data from the same study population, we extracted data only from cognitive tests that provided a unique contribution. In deciding which of the duplicate data to include, we selected the largest sample size. Although we searched for studies published in English, Spanish and Portuguese, only studies published in English met the eligibility criteria.

**Fig. 1:**
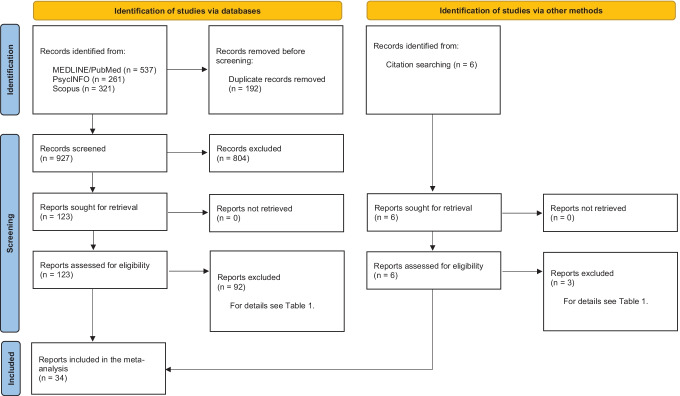
Flow chart of study selection

Figure 1 displays the study selection process during the second systematic review. We analyzed the full text of 129 articles deemed potentially eligible, of which 95 were excluded for the reasons described in Table 1. The included studies involved a variety of different neuropsychological tests that varied in the direction (interpretation) of the test score. For example, higher scores in the Trail Making Test (TMT) – Part A indicate poorer visual perception. In contrast, higher scores in Letter-Number Sequencing reflect higher working memory capacity and thus better performance. Therefore, prior to undertaking the meta-analysis, care was taken to align the directions of the average scores.

**Table 1:** Reasons for excluding studies from the meta-analysis

**Reasons**	***n***
Contained only duplicate data	11
Lack of cognitive assessment	14
Lack of control group without a family history of LOAD	45
Lack of required statistics to calculate effect sizes	9
Included participants with age-associated memory impairment	1
Included participants with mild cognitive impairment	1
Intermixed first and second-degree relatives	2
Intermixed first-degree relatives of individuals with LOAD-type and other dementia-type	2
Intermixed participants with and without LOAD-affected first-degree relatives	6
Could not determine the direction of the average scores	1
Unclear whether only LOAD or also early-onset Alzheimer's disease relatives were included	3

*LOAD* late-onset Alzheimer's disease

### Quality Assessment of Individual Studies

To assess the risk of quality-related bias within studies, we used the checklists for analytical cross-sectional, case–control, and cohort studies from the Joanna Briggs Institute (JBI) Critical Appraisal Tools (Moola et al., 2020). These checklists consist of questions evaluating concepts such as selection criteria, confounding factors, and measurement of exposure. For each question, a categorical outcome was allotted (yes, no, unclear, or not applicable). Regarding the criteria for determining risk of bias in individual studies, we adopted the following cut-off values. High risk of bias for studies with 49% or greater "yes" responses, moderate risk of bias for studies with 50% to 69% "yes" responses, and low risk of bias for studies with 70% or greater "yes" responses. These cut-off values have been adopted in previous meta-analytical reviews (Polmann et al., 2019; Sampaio et al., 2019). The first and the second authors critically appraised the quality of each included study against the criteria, and any discrepancies were resolved through discussion.

### Data Extraction and Coding

We coded data from each included study on demographic variables (e.g., age and years of education), APOE ε4 status (e.g., carriers vs. non-carriers), type of first-degree relative (e.g., offspring), characteristics of the study (e.g., authors, sample size, location), and the statistics from each cognitive test. In the Supplementary Online Content, Table S1 lists the included cognitive tests and specifies the relevant cognitive domain, Table S2 summarizes the individual effect sizes estimated according to the cognitive test, and Table S3 synthesizes age characteristics and percentage of females for first-degree relatives and controls. When the age range for each group was not documented in the original study, we listed the standard deviation in Table S3. In addition, for the purposes of this meta-analytic review, we assigned an age category to each group based on classifying study participants as young (< 40 years), middle-aged (40–65 years), and older (> 65 years) adults. This allowed us to consider the potential influence of age on cognitive dysfunction in first-degree relatives. Note that a predominance of studies included in this meta-analysis investigated first-degree relatives and matched controls aged 40–65 years (e.g., Abulafia et al., 2017; Sanchez et al., 2017). The first author extracted all relevant data from the included studies, and the second author reviewed and double-checked all extracted information. In addition to the Supplementary Online Content, spreadsheets containing the extracted data are openly available at https://osf.io/zjrxd/.

Note that we followed Strauss et al. (2006) as closely as possible to classify each cognitive test according to a primary domain of neuropsychological functioning (e.g., language, executive function). Given that TMT assesses a wide variety of cognitive processes (Salthouse, 2011; Strauss et al., 2006), we classified TMT Part A as a visual perception test and Part B as an executive function test. Several studies included in the meta-analysis provided separate scores for immediate and delayed memory recall. Thus, we included the former in the short-term (STM) or immediate memory (IM) domain and the latter in the long-term memory (LTM) domain, in addition to labeling both cognitive domains according to the modality of presentation (verbal vs. visuospatial). Separation of STM or IM verses LTM tests was important because LTM impairment is the main prominent cognitive symptom in the early stages of LOAD (Gallagher & Koh, 2011) and hence may be particularly affected in first-degree relatives. In relation to Bloss et al. (2008) finding evidence that school children (aged 11–16 years) with both a family history of LOAD and APOE ɛ4 genotype show significantly poorer scholastic achievement and inferior performance on cognitive tests (e.g., California Achievement Test and Rey-Osterrieth Complex Figure Test) compared to children without these risk factors, premorbid intelligence was deemed an important cognitive domain to be investigated in the current quantitative review.

### Statistical Analysis

To address the within-study effect size dependence, we fitted a three-level structural equation modeling meta-analysis with the *metaSEM* (Cheung, 2015) package in R (R Core Team, 2018). All R Studio scripts are available online (https://osf.io/zjrxd/). The effect size index for the current meta-analysis was Hedges' *g*, an estimator suitable for meta-analyses that include studies with small sample sizes (*n* < 20) (Hedges & Olkin, 1985). Hedges' *g* was interpreted according to the criteria of Cohen (1988), where ≈0.20 constitutes a small effect, ≈0.50 a medium effect, and ≥ 0.80 a large effect. In this quantitative synthesis, negative *g*-values indicate that first-degree relatives of individuals with LOAD had worse performance compared to controls. To account for the percentage of total variance within and between-studies due to heterogeneity not explained by sample error, in addition to τ^2^ that provides an absolute amount of effect size dispersion, we also employed *I*^2^ as a measure of inconsistency. Benchmarks proposed by Higgins et al. (2003) were followed to interpret *I*^2^ as ≈25%, small, ≈50%, moderate, and ≈75%, high. Since we carried out a three-level meta-analysis, we reported τ^2^_2_ and *I*^2^_2_ for the within-study variance (level 2) as well as τ^2^_3_ and *I*^2^_3_ for the between-study variance (level 3). Effect sizes deemed as outliers according to two-sided Grubbs' tests were excluded from the meta-analysis (see Table S2 note in the Supplementary Online Content).

We initially pooled all included effect sizes to provide an overall index of cognitive functioning of first-degree relatives compared to controls, and then we conducted several subgroup analyses to investigate the relevance of domain-specific indices of cognitive performance (e.g., executive functions, language), APOE ε4 status (e.g., carriers vs. non-carriers), type of first-degree relative (e.g., offspring), age category (e.g., middle-aged vs. middle-aged and older adults), and risk of bias in individual studies. Note that the fitted three-level subgroup analyses combined the subgroup weighted means and modelled the between-subgroup variance to control for studies contributing multiple effect sizes within the subgroups investigated. Regarding methodological quality, only one study (Rice et al., 2003) was judged as having high risk of bias (i.e., low methodological quality), and hence the subgroup analysis on risk of bias included only studies with low or moderate risk (i.e., high or moderate methodological quality). Similarly, we did not consider the study by Del Cerro et al. (2020) in the subgroup analysis on age category because this was the only study classified into the category young and middle-aged.

Univariate meta-regressions explored potential moderating effects of first-degree relative group demographic data (age, education, scores on MMSE, and percentage of females), whereas a multivariate meta-regression investigated the influence of cognitive test type on effect size heterogeneity. Since the preference for publishing studies with statistically significant results is the primary source of publication bias in meta-analyses (Button et al., 2016), we investigated publication bias using Egger's regression test and the three-parameter selection model (3PSM; Coburn & Vevea, 2019; Vevea & Hedges, 1995), which yields a likelihood-ratio indicating whether studies in a specific interval of significance (e.g., *p* < 0.05) were more likely to be published. For drawing a funnel plot, we first pooled multiple effect sizes from the same study using a three-level approach (for details, see R script “Effect Sizes for Each Study” available at https://osf.io/zjrxd/), thus only one effect size for each study was considered in the publication bias analysis.

## Results

### Characteristics of Included Studies

The overall meta-analysis included 4,086 first-degree relatives of individuals with LOAD (group mean age, mean = 57.40, *SD* = 4.71, range = 50–70) and 2,388 controls without family history of LOAD (group mean age, mean = 58.43, *SD* = 5.69, range = 49–76). Two-sided Grubb’s tests did not identify any statistically significant outlier in group mean age distribution for first-degree relatives (*G* = 3.07, *p* = 0.428) or controls (*G* = 3.52, *p* = 0.079). As indicated in Table S3, only 4 studies (Berti et al., 2011; Head et al., 2017; Jonaitis et al., 2015; Rice et al., 2003) documented the exact age ranges for first-degree relatives and controls. Out of 34, 13 (38.24%) studies (Abulafia et al., 2017, 2019a, b; Fleisher et al., 2005; Green & Levey, 1999; Johnson et al., 2006, 2018; La Rue et al., 2008; Mason et al., 2017; Rajah et al., 2017; Ravona-Springer et al., 2020; Sanchez et al., 2017; Sanchez-Benavides et al., 2016) included only middle-aged individuals (40–65 years), 12 (35.29%) studies (Aschenbrenner et al., 2016; Bassett et al., 2006; Berti et al., 2011; Debette et al., 2009; Fladby et al., 2017; Hazlett et al., 2015; Head et al., 2017; La Rue et al., 1996; Miller et al., 2005; Rice et al., 2003; Smith et al., 2010; Yassa et al., 2008) intermixed middle-aged and older (> 65 years) adults, four (11.76%) studies (Donix et al., 2010; Jonaitis et al., 2015; Mosconi et al., 2011, 2012) intermixed young (< 40 years), middle-aged, and older participants, one (2.94%) study intermixed young and middle-aged individuals (Del Cerro et al., 2020), and four (11.76%) studies (Bendlin et al., 2010; La Rue et al., 1995; Smith et al., 2002, 2005) did not provide sufficient information (no age ranges specified) to ascertain the probable age category of the participants (see Table S3 notes for details). Table 2 shows the demographic data and moderator variables analyzed in this quantitative synthesis. Twenty-four studies (70.5%) were conducted in the United States, four (11.8%) in Argentina, two (5.9%) in Spain, and four (11.8%) in other countries (Canada, Israel, Norway, and the United Kingdom). Only two studies (Bassett et al., 2006; Yassa et al., 2008) included relatives (offspring) of autopsy-confirmed LOAD cases.

**Table 2:** Demographic data and potential moderators

**Study**	**Location**	**Relative Type**	**Study**	**MMSE**	**MMSE**	**Age**	**Age**	**Edu**	**Edu**	**APOE ɛ4**	**APOE ɛ4**
			**Design**	**Controls**	**Rel**	**Controls**	**Rel**	**Controls**	**Rel**	**Controls**	**Relatives**
Abulafia et al. (2017)	Argentina	Offspring	Cross-sectional	29.30	28.90	54.20	53.80	17.10	16.30	—	—
Abulafia et al. (2019a, b)	Argentina	Offspring	Cross-sectional	—	—	52.21	54.59	17.64	17.16	—	—
Abulafia et al. (2019a, b)	Argentina	Offspring	Cross-sectional	29.50	28.74	51.22	55.26	17.65	16.96	—	—
Aschenbrenner et al. (2016)	USA	Offspring	Cross-sectional	29.30	29.20	66.00	64.00	16.40	16.30	Negative	Negative
Bassett et al. (2006)	USA	Offspring	Cross-sectional	27.80	28.20	62.68	62.01	16.10	15.31	Mixed	Mixed
Bendlin et al. (2010) – APOE ɛ4^+^	USA	Offspring	Cross-sectional cohort	29.50	29.50	55.40	57.50	16.90	16.30	Positive	Positive
Bendlin et al. (2010) – APOE ɛ4^−^	USA	Offspring	Cross-sectional cohort	29.40	29.50	58.40	57.40	16.70	16.10	Negative	Negative
Berti et al. (2011) – Maternal FH	USA	Offspring	Cross-sectional cohort	29.40	29.60	65.50	63.00	16.40	17.20	Mixed	Mixed
Berti et al. (2011) – Paternal FH	USA	Offspring	Cross-sectional cohort	29.40	29.60	65.50	62.90	16.40	16.90	Mixed	Mixed
Debette et al. (2009)	USA	Offspring	Prospective cohort	—	—	57.00	62.70	—	—	Mixed	Mixed
Del Cerro et al. (2020)	Spain	Offspring	Cross-sectional	29.61	28.87	53.90	50.36	17.47	17.75	—	—
Donix et al. (2010)	USA	Offspring	Cross-sectional	29.20	29.30	61.00	63.50	16.30	16.80	Mixed	Mixed
Fladby et al. (2017)	Norway	Any firs-degree	Cross-sectional	29.00	30.00	63.20	58.90	—	—	Mixed	Mixed
Fleisher et al. (2005)	USA	Any first-degree	Cross-sectional	29.00	29.80	60.10	60.70	16.90	16.20	Negative	Positive
Green and Levey (1999)	USA	Offspring	Case–control	—	—	55.40	55.50	15.10	14.40	—	Mixed
Hazlett et al. (2015)	USA	Any first-degree	Cross-sectional	28.75	29.17	69.23	66.11	14.88	15.75	—	—
Head et al. (2017)	USA	Offspring	Cross-sectional cohort	29.50	29.40	58.00	58.00	16.00	16.00	Mixed	Mixed
Johnson et al. (2018) – APOE ɛ4^−^	USA	Offspring	Prospective cohort	—	—	56.00	54.00	—	—	Negative	Negative
Johnson et al. (2018) – APOE ɛ4^+^	USA	Offspring	Prospective cohort	—	—	57.00	53.00	—	—	Positive	Positive
Johnson et al. (2018) – APOE ɛ4^−^	USA	Offspring	Prospective cohort	—	—	54.75	53.87	16.25	15.84	Negative	Negative
Johnson et al. (2018) – APOE ɛ4^+^	USA	Offspring	Prospective cohort	—	—	55.80	54.53	17.30	15.97	Positive	Positive
Johnson et al. (2006) – APOE ɛ4^−^	USA	Offspring	Cross-sectional cohort	—	—	54.75	53.87	16.25	15.84	Negative	Negative
Johnson et al. (2006) – APOE ɛ4^+^	USA	Offspring	Cross-sectional cohort	—	—	55.80	54.53	17.30	15.97	Positive	Positive
Jonaitis et al. (2015)	USA	Offspring	Prospective cohort	—	—	56.60	53.30	—	—	Mixed	Mixed
La Rue et al. (1995)	USA	Any first-degree	Cross-sectional cohort	—	—	58.50	55.10	15.10	14.60	—	—
La Rue et al. (1996)	USA	Any first-degree	Cross-sectional cohort	—	—	61.90	62.90	15.80	14.50	—	—
La Rue et al. (2008)	USA	Offspring	Cross-sectional cohort	—	—	55.70	52.37	16.57	16.00	—	—
Mason et al. (2017)	USA	Offspring	Cross-sectional	29.80	29.90	52.80	53.10	18.30	16.40	Mixed	Mixed
Miller et al. (2005)	USA	Any first-degree	Prospective cohort	—	—	63.67	64.33	15.96	15.75	Negative	Positive
Mosconi et al. (2011) – Paternal FH	USA	Offspring	Cross-sectional cohort	29.40	29.80	56.00	56.00	18.00	18.00	Mixed	Mixed
Mosconi et al. (2011) – Maternal FH	USA	Offspring	Cross-sectional cohort	29.40	29.40	56.00	55.00	18.00	17.00	Mixed	Mixed
Mosconi et al. (2012) – Paternal FH	USA	Offspring	Case–control	29.40	29.70	63.00	58.00	16.00	17.00	Mixed	Mixed
Mosconi et al. (2012) – Maternal FH	USA	Offspring	Case–control	29.40	29.70	63.00	60.00	16.00	16.00	Mixed	Mixed
Rajah et al. (2017) – APOE ɛ4^−^	Canada	Any first-degree	Cross-sectional	—	—	49.27	51.43	15.46	15.64	Negative	Negative
Rajah et al. (2017) – APOE ɛ4^+^	Canada	Any first-degree	Cross-sectional	—	—	49.27	51.91	15.46	14.92	Positive	Positive
Ravona-Springer et al. (2020)	Israel	Offspring	Cross-sectional cohort	29.18	29.23	56.42	54.55	16.80	16.39	Mixed	Mixed
Rice et al. (2003)	UK	Any first-degree	Cross-sectional cohort	28.00	27.00	76.00	70.00	11.00	10.00	Mixed	Mixed
Sanchez et al. (2017)	Argentina	Offspring	Cross-sectional	29.55	28.95	51.80	54.86	17.74	17.52	—	—
Sanchez-Benavides et al. (2016)	Spain	Offspring	Prospective cohort	28.96	29.12	54.62	52.59	13.81	14.06	Mixed	Mixed
Smith et al. (2002)	USA	Any first-degree	Cross-sectional cohort	29.10	29.00	53.20	52.00	15.30	14.80	Negative	Positive
Smith et al. (2010)	USA	Any first-degree	Cross-sectional cohort	29.10	29.30	68.10	57.70	15.90	15.70	Negative	Positive
Smith et al. (2005)	USA	Any first-degree	Prospective cohort	28.80	29.10	54.70	53.50	14.50	15.60	Negative	Positive
Yassa et al. (2008)	USA	Offspring	Cross-sectional	27.70	28.40	62.50	61.20	14.10	15.70	Negative	Mixed

*Rel.* relatives, *Edu.* Education, *FH* family history of Alzheimer's disease, *APOE ε4* ε4 allele of the apolipoprotein E (^+^, positive; ^−^, negative), *USA* United States, *UK* United Kingdom, *MMSE* Mini-Mental State Examination

Dashes (—) indicate information not provided

Only Rice et al. (2003) investigated only siblings, thus we documented as any first-degree relative for the purposes of the subgroup analyses

### Cognitive Functioning and Family History of Late-onset Alzheimer's Disease

**Fig. 2:**
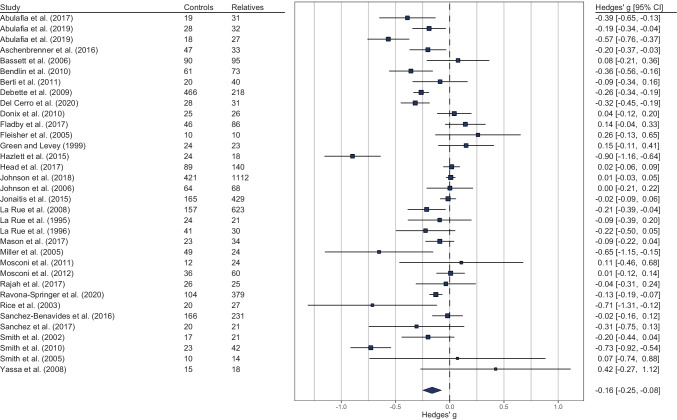
Forest plot illustrating for each study the estimated effect size and its 95% confidence interval (represented by the dark blue horizontal line). Effect sizes less than zero reflect worse cognitive performance in first-degree relatives of individuals with late-onset Alzheimer's disease compared to controls. The diamond summarizes the overall effect size. Group mean ages ranged from 50 to 70 in first-degree relatives and from 49 to 76 in the control groups

Overall, first-degree relatives showed significantly worse cognitive performance compared to controls (*g* = -0.16, 95% CI [-0.25, -0.08], *p* < 0.001), as illustrated in Fig. 2. Heterogeneity was not evident at level 2 (τ^2^_2_ = 0.00; *I*^2^_2_ = 0.00). However, the medium-to-large amount of heterogeneity at level 3 (τ^2^_3_ = 0.05; *I*^2^_3_ = 63.10) indicated that 63% of the observed variance comes from differences in effect sizes across studies. The subgroup analyses in Table 3 show cognitive domain had a moderating effect on between-group differences (*χ*^2^ (9) = 21.66, *p* = 0.010), accounting for 9.21% of the effect size variance at level 3 (*R*^2^_3_ = 0.0921). Specifically, first-degree relatives had significantly worse performance in executive functions (*g* = -0.17, 95% CI [-0.26, -0.07], *p* < 0.001), language (*g* = -0.28, 95% CI [-0.45, -0.10], *p* = 0.002), verbal IQ (*g* = -0.15, 95% CI [-0.27, -0.03], *p* = 0.012), verbal LTM (*g* = -0.17, 95% CI [-0.27, -0.07], *p* < 0.001) and STM or IM (*g* = -0.16, 95% CI [-0.26, -0.06], *p* = 0.002), and visuospatial LTM (*g* = -0.24, 95% CI [-0.46, -0.03], *p* = 0.028). First-degree relatives and controls did not differ in performance IQ (*g* = -0.005, 95% CI [-0.15, 0.14], *p* = 0.950), premorbid intelligence (*g* = -0.24, 95% CI [-0.49, 0.01], *p* = 0.060), visual perception (*g* = -0.03, 95% CI [-0.21, 0.15], *p* = 0.720), and visuospatial STM or IM (*g* = -0.22, 95% CI [-0.69, 0.25], *p* = 0.362).

**Table 3:** Subgroup effects on performance differences between first-degree relatives of individuals with Alzheimer's disease and controls

**Variables**	**Studies (*n*)**	**Effects Sizes (*n*)**	**Hedges'** ***g*** **[95% CI]**	***p*** **value**	***R***^**2**^_**2**_	***R***^**2**^_**3**_	**Likelihood-Ratio Test**
APOE ɛ4 Status	26	162	—	—	<.0001	.2817	χ^2^ (2) = 8.31, *p* = .016
Mixed (Carriers and Non-Carriers)	16	101	-0.04 [-0.13, 0.05]	.348	—	—	—
Carriers	9	38	-0.24 [-0.38, -0.11]	< .001	—	—	—
Non-Carriers	5	23	-0.14 [-0.28, -0.01]	.036	—	—	—
Cognitive Domain	34	218	—	—	<.0001	.0921	χ^2^ (9) = 21.66, *p* = .010
Executive Functions	18	61	-0.17 [-0.26, -0.07]	< .001	—	—	—
Language	9	13	-0.28 [-0.45, -0.10]	.002	—	—	—
Performance Intelligence Quotient	8	15	-0.005 [-0.15, 0.14]	.950	—	—	—
Premorbid Intelligence	6	6	-0.24 [-0.49. 0.01]	.060	—	—	—
Verbal Intelligence Quotient	11	17	-0.15 [-0.27, -0.03]	.012	—	—	—
Verbal Long-Term Memory	19	40	-0.17 [-0.27, -0.07]	< .001	—	—	—
Verbal Short-Term Memory	17	37	-0.16 [-0.26, -0.06]	.002	—	—	—
Visual Perception	11	16	-0.03 [-0.21, 0.15]	.720	—	—	—
Visuospatial Long-Term Memory	6	7	-0.24 [-0.46, -0.03]	.028	—	—	—
Visuospatial Short-Term Memory	5	6	-0.22 [-0.69, 0.25]	.362	—	—	—
Relative Type	34	218	—	—	<.0001	.1017	χ^2^ (1) = 2.80, *p* = .094
Any First-Degree Relative	11	46	-0.28 [-0.44, -0.12]	< .001	—	—	—
Offspring	23	172	-0.12 [-0.22, -0.02]	.015	—	—	—
Age Category^a^	29	185	—	—	<.0001	.0499	χ^2^ (2) = 2.84, *p* = .242
Middle-aged (40–65 years)	13	96	-0.12 [-0.26, 0.02]	.081	—	—	—
Middle-aged and older (40^+^ years)^b^	13	61	-0.23 [-0.37, -0.09]	.002	—	—	—
Young, middle-aged, and older	3	28	0.04 [-0.26, 0.34]	.800	—	—	—
Risk of Bias in Individual Studies	33	217	—	—	<.0001	.0011	χ^2^ (1) = 0.00, *p* = .956
Low	23	135	-0.15 [-0.26, -0.05]	.004	—	—	—
Moderate	10	82	-0.16 [-0.31, -0.01]	.042	—	—	—

*APOE ε4* ε4 allele of the apolipoprotein E gene, *R*^2^_2_ the ratio of variance explained by the model in level 2 (within-study variance), *R*^2^_3_  the ratio of variance explained by the model in level 3 (between-study variance)

Dashes (—) indicate not applicable

^a^The moderating effect of age category remained non-significant (χ^2^ (1) = 1.09, *p* = .297) when considering only middle-aged vs. middle-aged and older first-degree relatives

^b^Jonaitis et al. (2015) included young, middle-aged, and older controls (36-68 years) but only middle-aged and older first-degree relatives (40-67 years)

### APOE ɛ4, Relative Type, Demographic Data, Age Category, and Cognitive Test Type

Table 3 shows more significant overall cognitive dysfunction in first-degree relatives who are APOE ɛ4 carriers (*g* = -0.24, 95% CI [-0.38, -0.11], *p* < 0.001) compared to non-carriers (*g* = -0.14, 95% CI [-0.28, -0.01], *p* = 0.036) or mixed samples (*g* = -0.04, 95% CI [-0.13, 0.05], *p* = 0.348). In addition, statistically significant differences among the three subgroups were identified (*χ*^2^ (2) = 8.31, *p* = 0.016) such that APOE ɛ4 status of the first-degree relatives accounted for 28.17% of the between-study variance (*R*^2^_3_ = 0.2817). Although offspring showed a significant effect size (*g* = -0.12, 95% CI [-0.22, -0.02], *p* = 0.015) seemingly smaller than samples that included any first-degree relatives (*g* = -0.28, 95% CI [-0.44, -0.12], *p* < 0.001), there was not enough evidence to reject the null hypothesis of equal effect sizes in the two subgroups (*χ*^2^ (1) = 2.80, *p* = 0.094). Similarly, samples intermixing middle-aged and older first-degree relatives appeared to exhibit a larger dysfunction effect size (*g* = -0.23, 95% CI [-0.37, -0.09], *p* = 0.002) compared to those including only middle-aged individuals (*g* = -0.12, 95% CI [-0.26, 0.02], *p* = 0.081). However, there was no evidence to support the hypothesis that age category explains the variation in effect sizes (χ^2^ (2) = 2.84, *p* = 0.242). The moderating effect of age category remained non-significant (χ^2^ (1) = 1.09, *p* = 0.297) when considering only middle-aged verses middle-aged and older first-degree relatives.

Table 4 shows there was no statistically significant effect of the first-degree-relative mean age (*β* = -0.012, 95% CI [-0.030, 0.006], *p* = 0.209), mean years of education (*β* = 0.019, 95% CI [-0.059, 0.097], *p* = 0.627), Mini-Mental State Examination (MMSE) mean scores (*β* = 0.196, 95% CI [-0.022, 0.413], *p* = 0.077), or percentage of females (*β* = -0.006, 95% CI [-0.012, 0.000], *p* = 0.061) on the overall cognitive performance difference of first-degree relatives against controls. Table S4 in the Supplementary Online Content shows that cognitive test type was the primary source of heterogeneity across effect sizes (*χ*^2^ (42) = 90.31, *p* < 0.001) in the fitted model, such that cognitive test type accounted for 65% of the between-study variance (*R*^2^_3_ = 0.6499). This result is not surprising given the diversity of cognitive test types considered in this multivariate meta-regression (see Table S4).

**Table 4:** Univariate meta-regression analyses on demographic data of the first-degree relative groups

**Covariates**	**Studies (*n*)**	**Effect Sizes (*n*)**	***β*-** **Coefficient [95% CI]**	***p*** **value**	***R***^**2**^_**2**_	***R***^**2**^_**3**_
Age (years)	34	218	-0.012 [-0.030, 0.006]	.209	<.0001	.0385
Education (years)	30	189	0.019 [-0.059, 0.097]	.627	.0094	<.0001
MMSE	23	156	0.196 [-0.022, 0.413]	.077	<.0001	.1938
% Females	30	199	-0.006 [-0.012, 0.000]	.061	<.0001	.0562

*MMSE* Mini-Mental State Examination, *R*^2^_2_ the ratio of variance explained by the model in level 2 (within-study variance), *R*^2^_3_  the ratio of variance explained by the model in level 3 (between-study variance)

### Risk of Bias in Individual Studies

The systematic review yielded 26 cross-sectional (Abulafia et al., 2017, 2019a, b; Aschenbrenner et al., 2016; Bassett et al., 2006; Bendlin et al., 2010; Berti et al., 2011; Del Cerro et al., 2020; Donix et al., 2010; Fladby et al., 2017; Fleisher et al., 2005; Hazlett et al., 2015; Head et al., 2017; Johnson et al., 2006; La Rue et al., 1995, 1996, 2008; Mason et al., 2017; Mosconi et al., 2011; Rajah et al., 2017; Ravona-Springer et al., 2020; Rice et al., 2003; Sanchez et al., 2017; Smith et al., 2002, 2010; Yassa et al., 2008), two case–control (Green & Levey, 1999; Mosconi et al., 2012), and six prospective cohort (Debette et al., 2009; Johnson et al., 2018; Jonaitis et al., 2015; Miller et al., 2005; Sanchez-Benavides et al., 2016; Smith et al., 2005) studies. In the Supplementary Online Content, Tables S5, S6, and S7 summarize the results regarding the assessment of risk of bias for each included study according to the respective research design. Overall, only one cross-sectional study was judged as having high risk of bias or low quality (score ≤ 49%), whereas one case–control and nine cross-sectional studies (29.41% of the included studies) were deemed as having moderate risk or quality (score 50-69%). On the other hand, six prospective cohort, one case–control, and 16 cross-sectional studies (67.65% of the included studies) met the criteria for low risk of bias or high methodological quality (score ≥ 70%). Importantly, as illustrated in Table 3, the subgroup analysis on risk of bias in individual studies showed that studies judged as having low (*g* = -0.15, 95% CI [-0.26, -0.05], *p* = 0.004) or moderate (*g* = -0.16, 95% CI [-0.31, -0.01], *p* = 0.042) risk yielded very similar effect sizes, and the two subgroups did not differ (*χ*^2^ (1) = 0.003, *p* = 0.956). These results indicate that the methodological quality of the included studies showed no association with cognitive performance differences between first-degree relatives and controls.

### Risk of Publication Bias

**Fig. 3:**
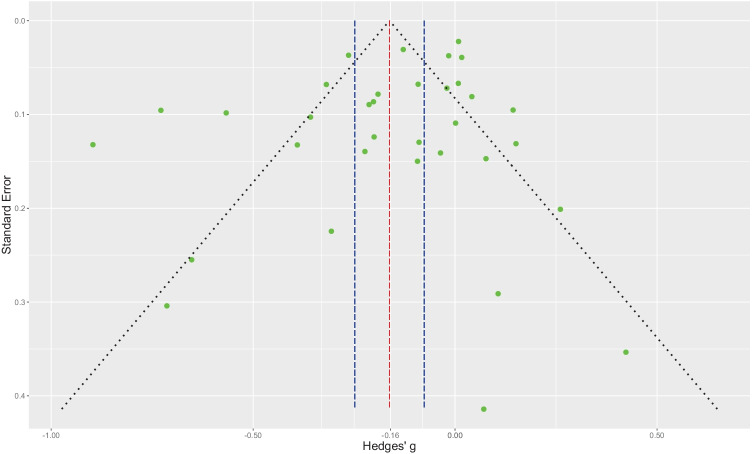
Funnel plot for publication bias for the 34 studies included in the meta-analysis. Green symbols represent the distribution of the estimated effect size for each study. The dashed red line depicts the pooled effect size, whereas the dashed blue lines demarcate its 95% confidence interval. Both Egger’s regression test (*z* = -0.23, *p* = .820) and the 3PSM likelihood-ratio test (χ^2^(1) = 3.44, *p* = .063) indicated no significant publication bias in the current meta-analysis

Figure 3 shows a funnel plot for publication bias analysis and illustrates the distribution of the pooled effects from the 34 studies included in this quantitative review. The fairly symmetrical distribution of the data points on both sides of the funnel indicates no significant publication bias. In addition, both Egger’s regression test (*z* = -0.23, *p* = 0.820) and the 3PSM likelihood-ratio test (χ^2^ (1) = 3.44, *p* = 0.063) indicate the current meta-analysis seems robust to publication bias.

## Discussion

To our knowledge, this is the first meta-analysis to quantify the impact of family history of LOAD on cognition, summarizing 218 effect sizes from 34 empirical studies. The results provide compelling evidence that first-degree relatives show a mild but robust amount of overall cognitive dysfunction compared to controls without LOAD-affected relatives. Cognitive deficits in first-degree relatives were evident in executive functions, language, verbal IQ, verbal and visuospatial LTM, and verbal STM or IM. These outcomes indicate that, compared to controls without a family history of LOAD, first-degree relatives have higher chances of obtaining lower scores on neuropsychological measures across multiple cognitive domains. One plausible explanation for these findings relates to altered biomarkers in probands of LOAD-affected individuals. For instance, previous studies have indicated that unaffected offspring of individuals with LOAD show morphological and metabolic brain changes that resemble the preclinical manifestations of LOAD-related pathology (Dubois et al., 2016), including increased global brain atrophy rates (Debette et al., 2009), reduced medial temporal lobe activation (Donix et al., 2010; Johnson et al., 2006), higher levels of beta-amyloid deposition (Clark et al., 2016; Duarte-Abritta et al., 2018), and decreased gray matter volume (Berti et al., 2011; Honea et al., 2010). On the other hand, the lack of significant group differences in premorbid intelligence and visuospatial STM or IM, and especially the near null effects in performance IQ and visual perception, suggest that having a family history of LOAD does not seem to be associated with significant decline in these domains. Alternatively, first-degree relatives may exhibit distinct patterns of cognitive dysfunction related to phenotypic differences in LOAD (Carrasquillo et al., 2014; Ferreira et al., 2020; Snowden et al., 2007; Vogel et al., 2021). For example, recent research indicated that the limbic-predominant phenotype is strongly associated with the amnestic presentation of the disease (e.g., LTM dysfunction), whereas the posterior phenotype is characterized by visuospatial or perceptual abnormalities (Vogel et al., 2021).

Notably, subgroup analyses revealed that the APOE ɛ4 genotype moderates performance differences between first-degree relatives and controls without a family history of LOAD, which makes sense given that the APOE ɛ4 genotype is the most replicated risk factor for LOAD in genetics studies (Cacabelos, 2003; Yang et al., 2021). Specifically, relative groups documented as ɛ4 carriers exhibited more significant dysfunction in cognition (*g* = -0.24) compared to relative groups documented as non-ɛ4 carriers (*g* = -0.14). This finding is consistent with preliminary research (Debette et al., 2009; Tsai et al., 2021) demonstrating that first-degree relatives with both risk factors (APOE ɛ4 genotype and a family history of LOAD) are more likely to present with deficits in cognition (e.g., executive dysfunction and verbal and visuospatial LTM difficulties). Evidence also suggests that first-degree relatives with both risk factors exhibit greater beta-amyloid deposition (Yi et al., 2018), higher brain atrophy rates (Debette et al., 2009), and reduced gray matter volume (Ten Kate et al., 2016) compared to those with only one risk factor. Nevertheless, the current systematic synthesis revealed that few studies on the topic document separate scores for ɛ4 carriers verses non-carriers. Hence, the lack of control for APOE ɛ4 status might help account for the contradictory findings from empirical studies on cognition of first-degree relatives of LOAD-affected individuals previously noted in the introduction, and if factored in to analyses of cognitive domains, could potentially paint a different picture with regard to the domains that did not reach statistical significance. Moving forward from the current outcomes, a major challenge for future research on the topic is to determine the combined effects and parse out the unique contributions of APOE ɛ4 carriership and a family history of LOAD in profiling cognitive dysfunction in first-degree relatives. Importantly, the APOE ε4 effect on cognition reported here is based on a specific sample (first-degree relatives of LOAD-affected individuals) and hence our results do not apply to the general population of APOE ε4 carriers.

Although relative group mean age was not a significant moderator and the null hypothesis on the equality of effect sizes in the subgroup analysis on age category was not rejected, the dysfunction effect size for samples intermixing middle-aged (40–65 years) and older (> 65 years) first-degree relatives (*g* = -0.23, 95% CI [-0.37, -0.09], *p* = 0.002) was statistically significant and nearly twice the size of the dysfunction effect for samples including only middle-aged individuals (*g* = -0.12, 95% CI [-0.26, 0.02], *p* = 0.081). This suggests that the inclusion of a large percentage of middle-aged individuals in the studies analyzed here may have led to an overall smaller dysfunction effect size (*g* = -0.16, 95% CI [-0.25, -0.08], *p* < 0.001) than might be expected in older cohorts, thus calling into question the generalizability of the current findings. This conjecture seems in line with findings from a previous study noted in the introduction (Zeng et al., 2013), in which, compared to controls, family members of LOAD-affected individuals showed substantial differences on neuropsychological measures only quite late in life (70 or more years).

The effects of a family history of LOAD on cognition remain poorly understood. Cognitive dysfunction in first-degree relatives of AD-affected individuals has gained attention only in the last two decades. Figure 2 shows that out of 34 empirical works, only three studies (Green & Levey, 1999; La Rue et al., 1995, 1996) were published before the current century, and all of the studies were published within the past 30 years. As previously noted, LOAD-related neuropathological changes precede the clinical diagnosis of LOAD by many years, hence, an increasing number of studies has attempted to longitudinally follow cognitive changes and brain abnormalities in earlier first-degree relatives. In this meta-analytic review, some included studies were drawn from ongoing prospective studies, thus, follow-up research on these cohorts as they grow older is expected. This will allow investigation of cognitive dysfunction in older cohorts of first-degree relatives with a family history of LOAD.

## Implications

Findings from the current quantitative review may have important clinical and theoretical implications. LOAD is an age-dependent dementing disease with cognitive symptoms that appear after a lengthy period of evolving neuropathophysiological abnormalities, and thus the effect sizes for between-group differences in several cognitive domains reported here may assist in establishing sensitive cognitive markers for first-degree relatives. This assertion builds on previous empirical research indicating that impairments in cognitive abilities such as premorbid intelligence, memory, and language are deemed potential markers for future development of LOAD (Blacker et al., 2007; Chen et al., 2000; Rapp & Reischies, 2005; Yeo et al., 2011). Equally important, executive dysfunction can be detected in middle-aged offspring many years before the affected parent develops dementia (Debette et al., 2009; Eyigoz et al., 2020). Hence, developing cognitive-based interventions for first-degree relatives, especially APOE ɛ4 carriers, is a pressing need. In relation to this, recent randomized controlled trials have shown that cognitive training benefits individuals at the early stages of LOAD (Cavallo et al., 2016; Kang et al., 2019; Lee et al., 2013). To our knowledge, however, no study has addressed the potential benefit of such a therapeutic strategy in buffering against cognitive decline in unaffected first-degree relatives of LOAD-affected individuals. 

## Strengths and limitations

Notwithstanding the fact that only studies published in English met the eligibility criteria, we systematically searched for studies published in English, Portuguese, and Spanish, which is a procedure that minimizes the risk of language-related bias given that the inclusion of studies published in languages other than English is often neglected in meta-analyses (Sterne et al., 2001). Another important strength of the current research synthesis is the control for multiple publications involving the same or overlapping study populations, such that only a single effect size for each cognitive test from the included studies contributed to the main meta-analysis, thus limiting the influence of multiple outcomes involving the same individuals. However, this methodology did not preclude that multiple effect sizes involving the same individuals were included in the subgroup analyses (e.g., cognitive domain).

Several caveats of the current quantitative synthesis should be acknowledged. For example, a limitation worth noting relates to the lack of a systematic literature search for unpublished studies. Although the current meta-analysis seemed robust to publication bias, additional unpublished research could provide more data to increase the statistical power in the subgroup analyses. However, this necessarily comes with risks due to the lack of peer-review. Another limitation is that 70.5% of the included studies were conducted in the United States, which may introduce concern regarding the representativeness of the population with a family history of LOAD. In addition, the small number of studies for some of the variables included in the subgroup analyses (e.g., premorbid intelligence, visuospatial STM or IM, visuospatial LTM) may limit the interpretation of the respective outcomes and thus warrant confirmation through further research. Similarly, the absence of statistically significant between-subgroup differences in the subgroup analyses cannot be directly deemed as evidence of equal population effect sizes across the subgroups investigated because the statistical power of such analyses may be insufficient to detect small differences between the subgroups. Furthermore, because only one study (Rice et al., 2003) reported cognitive test results for siblings in isolation from offspring, we could not investigate cognitive profile differences between siblings and offspring.

## Conclusion

Findings across several cognitive domains indicate that differences in cognition are present in first-degree relatives of LOAD-affected individuals compared to controls, albeit some cognitive domains showed no substantial evidence of dysfunction. Notably, the outcomes suggest that the APOE ε4 allele plays a pivotal role in determining more significant cognitive difficulties in first-degree relatives. In addition to providing directions for future research, the current quantitative synthesis helps elucidate neuropsychological abnormalities associated with a family history of LOAD, pointing to the importance of exploring preventive interventions targeting cognitive decline in first-degree relatives of LOAD-affected individuals.
